# Effect of blood transfusion during cesarean section on postpartum hemorrhage in a tertiary hospital over a 4-year period

**DOI:** 10.1097/MD.0000000000023885

**Published:** 2021-01-22

**Authors:** Changqing Zhou, Li Zhang, Yang Bao, Ling Li, Ting Zhang, Xiyan Zhang, Chunling Wang

**Affiliations:** Department of Anesthesiology, Qilu Hospital of Shangdong University, 107 Wenhua Xi Road, Jinan, Shandong, China.

**Keywords:** placenta previa, postpartum hemorrhage, blood transfusion, uterine scar

## Abstract

Postpartum hemorrhage (PPH) is the leading cause of maternal morbidity and death worldwide. The history of cesarean section and the occurrence of placenta previa were significantly associated with the increase in blood transfusion. Therefore, to prevent PPH, it is important to understand the effect of blood transfusion during cesarean section on postpartum hemorrhage. The purpose of this study is to determine the cause of blood transfusion during cesarean section, especially large amounts of blood transfusion, and to take measures to reduce the blood demand caused by PPH with limited blood supply.

This study was a retrospective study of patients who underwent blood transfusion during cesarean section in Qilu Hospital of Shandong University (China) from January 2013 to December 2016. Red blood cell infusion ≥10 U during cesarean section was defined as massive blood transfusion. The study collected the demographics of pregnant women, obstetric characteristics and reasons for blood transfusions, as well as blood components and blood transfusion results. Multivariate regression analysis was performed for evaluating the risk factors of PPN.

From 2013 to 2016, a total of 587 patients received blood transfusions during cesarean section. The proportion of women receiving blood transfusion during cesarean section increased (from 3.21% to 7.40%, *P* < .001). The history of cesarean section (*P* = .005) and the occurrence of placenta previa were positively correlated with the increase in blood transfusion (*P* = .016). There were 72 cases of massive blood transfusion, accounting for 12.27% of blood transfusion patients. Among mass blood transfusions, 93.1% of cases had prior cesarean delivery, and placenta previa accounted for 95.8%. 19.4% of patients receiving massive blood transfusions underwent hysterectomy. There was no significant difference in maternal BMI and gestational age between the mass blood transfusion group and the non-mass blood transfusion group.

From 2013 to 2016, the demand for blood transfusion, especially the demand for massive blood transfusion, increased. Repeated cesarean section and placental previa combined with uterine scar are positively correlated with increased blood transfusion. Reducing the initial cesarean section should help reduce the massive blood transfusion caused by placenta previa with a history of cesarean section.

## 1 Introduction

Postpartum hemorrhage (PPH) is a leading cause of maternal mortality. All pregnant women with gestational age of >20 weeks are at risk of PPH.^[1]^ According to report of WHO on developing countries in 2007, maternal death rates are 1000 per 100,000 live births, of which PPH accounted for 60%.^[2]^ Importantly, the PPH rate is increased from 1.5% in 1999 to 4.1% in 2009 in Iraland.^[3]^ The risk factors for PPH include multiparous, obesity, repeated cesarean section, advanced maternal age, and pregnancy comorbidities such as pregnancy-induced hypertension.^[4–7]^

In China, family planning and one-child policy has been implemented for more than thirty years to control population growth, with some exceptions. As the population aging problem has intensified, each couple has been allowed to have 2 children since the end of 2013. Nevertheless, with the introduction of this policy, the increasing in births has led to blooming in the demand for obstetric services, as well as repeated pregnancy and incidence of pernicious placenta previa.^[8–10]^ Of note, intraoperative bleeding of pernicious placenta previa is rapid and massive, hemostasis is difficult, and large amounts of blood transfusion and sometimes hysterectomy are necessary to control bleeding. Moreover, PPH associated with pernicious placenta previa disease is extremely dangerous and may lead to serious complications such as hemorrhagic shock, disseminated intravascular coagulation (DIC) and metabolic acid-base disorder.^[11,12]^

Increasing the need for massive blood transfusion has been noticed clinically with impact of two-child policy in China. Some risk factors are associated with PPH, such as massive blood transfusion during cesarean section, pernicious placenta previa and combined gestational hypertension. Altering those modifiable factors might be able to reduce transfusions and PPH. The hypothesis of the study is that the previous cesarean delivery and uterine scar are associated with the demand of blood transfusion. The aim of our study was to analyze the changes in blood transfusion during cesarean section, and advocate the reduction of primary cesarean section to decrease adverse consequences caused by the history of cesarean section.

## 2 Material and methods

### 2.1 Study design and patients

This was a retrospective study of patients who received blood transfusion during cesarean section in Qilu Hospital of Shandong University between January 2013 and December 2016. The study was approved by the Ethics Committee of Qilu Hospital of Shandong University, China (No: 2017018).

Data was extracted from digital medical record management system of Qilu Hospital of Shandong University. According to clinical cases of blood transfusion during cesarean section, national version of the International Classification of Diseases (ICD)-10 diagnosis coding and Beijing version of the ICD-9 surgery code were used to confirm obstetric comorbidities. The inclusion criteria included infusion of any blood products during cesarean section, such as red blood cell, plasma, platelets and cryoprecipitate. An amount of red blood cell infusion **≥**10 U (1 U of red blood cell suspension was prepared by 200 ml of whole blood) was considered to be massive blood transfusion.

### 2.2 Data collection

The collected data included age of patients, gestational age, body mass index (BMI), gravidity, singleton/multiple pregnancy, history of hypertension, obstetrical pathologies, any other significant comorbidities, number of previous abortions, history of cesarean section or uterine surgery, clinical diagnosis, surgical time, anesthesia type, hospitalization, postoperative hospitalization, emergency vs. elective surgery, neonatal outcomes, intraoperative bleeding volume, infusion of erythrocytes, plasma, platelets and cryoprecipitate, preoperative and postoperative hemoglobin, platelets, prothrombin time (PT), activated partial thromboplastin time (APTT) and fibrinogen, patients with red blood cell infusion **≥**10 U, and indications for hysterectomy. The estimated blood loss based on surgical records was established from the suction device, gauze pad and vaginal bleeding.

### 2.3 Statistical analysis

Sample size analysis was carried out by one way ANOVA – power analysis was used to achieve a power of 0.80 at a α level of 0.05. Statistical analysis was conducted using SPSS 23.0 (IBM, Armonk, NY, USA). Continuous variables with normal distribution were presented as mean (standard deviation [SD]); and were analyzed using the ANOVA followed by Bonferronis post-hoc comparisons tests. Categorical data were presented as frequencies and were analyzed using the chi-square test. Two-sided *P* value <.05 was considered statistically significant.

## 3 Results

### 3.1 Characteristics of patients

A total of 9691 cases underwent cesarean section from 2013 to 2016, and 587 of them received blood transfusions were included. There was no difference in maternal age and maternal BMI. As noticed, the proportion of blood transfusion during cesarean section increased from 3.21% in 2013 to 7.40% in 2016 (*P* < .001). The gravidity and parity numbers and previous cesarean section rate had significant differences from *P* = .02 and *P* = .006 from 2013 to 2016 (Table 1).

**Table 1:** Maternal characteristics of cases underwent cesarean section accepted blood transfusion during 2013 to 2016.

	2013	2014	2015	2016	Total	*P*
Portion of blood transfusion (%)	3.21 (64/1994)	5.31 (141/2653)	7.84 (155/1977)	7.40 (227/3067)	6.1 (587/9691)	<.001
Maternal age, year						.055
<24	11 (17.2)	9 (6.4)	15 (9.7)	11 (4.8)	46 (7.8)	
24–29	19 (29.7)	61 (43.3)	57 (36.8)	83 (36.6)	220 (37.5)	
30–39	33 (51.6)	63 (44.7)	75 (48.4)	123 (54.2)	294 (50.1)	
≥40	1 (1.6)	8 (5.7)	8 (5.2)	10 (4.4)	27 (4.6)	
Gestational age in weeks						.218
<29	0 (0.0)	8 (5.7)	11 (7.1)	14 (6.2)	33 (5.6)	
29–34^+6^	18 (28.1)	26 (18.4)	46 (29.7)	62 (27.3)	152 (25.9)	
35–36^+6^	13 (20.3)	28 (19.9)	32 (20.6)	48 (21.1)	121 (20.6)	
≥37	33 (51.6)	79 (56.0)	66 (42.6)	103 (45.4)	281 (47.9)	
Body mass index (kg/m^2^)						.791
<28	23 (38.3)	46 (35.1)	57 (39.9)	89 (41.6)	215 (39.2)	
28–32.9	30 (50.0)	73 (55.7)	69 (48.3)	99 (46.3)	271 (49.5)	
≥33	7 (11.7)	12 (9.2)	17 (11.9)	26 (12.1)	62 (11.3)	
Gravidity and parity numbers						.020
0	20 (31.2)	25 (17.7)	26 (16.8)	34 (15.0)	105 (17.9)	
1	19 (29.7)	43 (30.5)	41 (26.5)	52 (22.9)	155 (26.4)	
≥2	25 (39.1)	73 (51.8)	88 (56.8)	141 (62.1)	327 (55.7)	
Fetus number						.226
1	59 (92.2)	127 (90.1)	144 (92.9)	217 (95.6)	547 (93.2)	
≥2	5 (7.8)	14 (9.9)	11 (7.1)	10 (4.4)	40 (6.8)	
Number of previous cesarean section						.006
0	40 (62.5)	70 (49.6)	69 (44.5)	85 (37.4)	264 (45.0)	
1	20 (31.2)	58 (41.1)	79 (51.0)	120 (52.9)	277 (47.2)	
≥2	4 (6.2)	13 (9.2)	7 (4.5)	22 (9.7)	46 (7.8)	
Combined Gestational hypertension	24 (37.5)	24 (17.0)	36 (23.2)	42 (18.5)	126 (21.5)	.005

### 3.2 Causes of blood transfusion

The common causes of blood transfusion during cesarean section included placenta previa, anemia, preeclampsia, placental abruption, acute fatty liver of pregnancy (AFLP), and other blood diseases (Table 2). During 2013 to 2016, there were minor changes in frequencies of anemia, placental abruption, AFLP, and blood diseases (all *P* > .05). The blood transfusion rate due to preeclampsia was high in 2013, but decreased in 2014, 2015, and 2016 (*P* = .02). The frequency of blood transfusion during cesarean section in patients with placenta previa increased from 29.69% in 2013 to 51.10% in 2016 (*P* = .02). Moreover, the proportion of patients with pernicious placenta previa among patients who received blood transfusion increased constantly from 9.38% to 32.6% (*P* < .001).

**Table 2:** Comparison of maternal complications and haematological features among parturients accepted blood transfusion during 2013 to 2016.

Variables	2013 n = 64	2014 n = 141	2015 n = 155	2016 n = 227	*P* value
Maternal Complications					
Placenta previa	19 (29.69)	59 (41.84)	74 (47.74)	116 (51.10)	.016
Pernicious placenta previa	6 (9.38)	26 (18.44)	38 (24.52)	74 (32.60)	<.001
Anemia	6 (9.38)	20 (14.18)	23 (14.84)	21 (9.25)	.275
Preeclampsia	18 (28.12)	17 (12.06)	24 (15.48)	31 (13.66)	.021
Placental abruption	2 (3.12)	5 (3.55)	3 (1.94)	7 (3.08)	.859
Acute fatty liver of pregnancy	7 (10.94)	13 (9.22)	9 (5.81)	14 (6.17)	.399
Other blood diseases	12 (18.75)	27 (19.15)	22 (14.19)	38 (16.74)	.687
RBC infusion					
≥18 U	1 (1.56)	4 (2.84)	7 (4.52)	11 (4.85)	.563
10–18 U	6 (9.38)	12 (8.51)	12 (7.74)	19 (8.37)	.983
<10 U	24 (37.5)	80 (56.74)	95 (61.29)	134 (59.03)	.009
Blood loss ≥1000 ml	13 (20.31)	41 (29.08)	52 (33.55)	79 (34.80)	.136
Hysterectomy	0	2 (1.4)	6 (3.9)	6 (2.6)	.300

### 3.3 Causes for massive blood transfusion

Causes for massive blood transfusion were presented in Table 3. Compared with patients received <10 U of red blood cells, those who received >10 U of red blood cells were tend to be elderly maternal, older (*P* = .001), or a higher gravidities and garities (*P* < .001), or a higher incidence of previous cesarean section (*P* < .001), or a lower occurrence of gestational hypertension (*P* < .001), or a higher incidence of placenta previa (*P* < .001), or a higher incidence of hysterectomy (*P* < .001), and or a higher incidence of general anesthesia (*P* = .004). The stratification of different age groups was classified as: <24 years old young women, 30 to 39 years old women and women ≥40 years old. However, according to the maternal age proposed by RCOG and ACOG, the evidence has not been confirmed, and the reason may not be reasonable. Compared to multiple pregnancy, singleton pregnancy showed a significantly higher rate for receiving red blood cells >10 U (*P* = .02, Table 4).

**Table 3:** Comparison of maternal characteristics and obstetric complications between massive blood transfusion and non-massive blood transfusion during 2013-2016.

	Massive blood transfusion	Non-massive blood transfusion	Total	
Variables	N = 72	N = 515	N = 587	*P* value
Maternal Age (years)				.001
<24	0 (0.0)	46 (8.9)	46 (7.8)	
24–29	20 (27.8)	200 (38.8)	220 (37.5)	
30–39	45 (62.5)	249 (48.3)	294 (50.1)	
≥40	7 (9.7)	20 (3.9)	27 (4.6)	
Gestational age (w)				.083
<29	8 (11.1)	25 (4.9)	33 (5.6)	
29–34^+6^	21 (29.2)	131 (25.4)	152 (25.9)	
35–36^+6^	16 (22.2)	105 (20.4)	121 (20.6)	
≥37	27 (37.5)	254 (49.3)	281 (47.9)	
Body mass index (kg/m^2^)				.157
<28	26 (39.4)	189 (39.2)	215 (39.2)	
28–32.9	37 (56.1)	234 (48.5)	271 (49.5)	
≥33	3 (4.5)	59 (12.2)	62 (11.3)	
Gravidity and parity numbers				<.001
0	1 (1.4)	104 (20.2)	105 (17.9)	
1	12 (16.7)	143 (27.8)	155 (26.4)	
≥2	59 (81.9)	268 (52.0)	327 (55.7)	
Fetus number				.089
1	71 (98.6)	476 (92.4)	547 (93.2)	
≥2	1 (1.4)	39 (7.6)	40 (6.8)	
Number of previous cesarean section				<.001
0	5 (6.9)	259 (50.3)	264 (45.0)	
1	46 (63.9)	231 (44.9)	277 (47.2)	
≥2	21 (29.2)	25 (4.9)	46 (7.8)	
Gestational hypertension	2 (2.8)	124 (24.1)	126 (21.5)	<.001
Placenta previa	69 (95.8)	210 (40.8)	279 (47.5)	<.001
Hysterectomy	14 (19.4)	0	14 (2.4)	<.001
CD surgery type				.098
Selective surgery	17 (23.6)	78 (15.1)	492 (83.8)	
Emergency surgery	55 (76.4)	437 (84.9)	95 (16.2)	
Anesthesia type				.004
General anesthesia	66 (91.7)	390 (75.7)	456 (77.7)	
Neuraxial anesthesia	6 (8.3)	125 (24.3)	131 (22.3)	

**Table 4:** Comparison of massive blood transfusion between singleton Pregnancy and multiple Pregnancy.

Pregnancy	RBC infusion <10U (n)	RBC infusion >10U^∗^ (n)
Singleton	302	71
multi	31	1

### 3.4 Hematological features and outcomes of coagulation function

Comparisons of blood components and outcome evaluation in patients with massive blood transfusion were shown in Table 5. There was no difference in the ratio of red blood cells to plasma transfusion, frequency of abnormal coagulation after transfusion, preoperative and postoperative platelets, and postoperative prothrombin time, activated partial thromboplastin time, fibrinogen, and hospitalization (*P* value >.05 for all) from 2013 to 2016. The frequencies of platelet infusion were of 14.3% in 2013 to 66.7% in 2016 (*P* = .01), using tranexamix acid (trans-4-amino-methyl-cyclohexane-carboxylic acid) of 0% in 2013 to 86.7% in 2016 (*P* < .001) and postoperative hemoglobin levels of 87.0 ± 14.9 g/L in 2013 to 103.4 ± 20.1 g/L in 2016 (*P* = .003).

**Table 5:** Hematological features for massive blood transfusion and outcomes after blood transfusion.

Patients with massive blood transfusion (n)	2013 n = 7	2014 n = 16	2015 n = 19	2016 n = 30	*P*
Red blood cells 1 U: plasma of 100 ml	1.06 ± 0.30	1.30 ± 0.47	1.27 ± 0.38	1.13 ± 0.31	.250
Platelet infusion (%)	1 (14.3)	5 (31.2)	6 (31.6)	20 (66.7)	.012
Used tranexamic acid (%)	0 (0)	1 (6.25)	18 (94.7)	26 (86.7)	<.001
Coagulation deficiency after blood transfusion(%)	3 (42.9)	6 (37.5)	9 (47.4)	12 (40.0)	.954
Postoperative platelets <50,000/dl (%)	1 (14.3)	1 (6.25)	2 (10.5)	2 (3.33)	.886
Postoperative platelets <100,000/dl and >50,000/dl (%)	1 (14.3)	2 (12.5)	5 (26.3)	7 (23.3)	.725
Postoperative hemoglobin (g/l)	87.0 ± 14.9	89.3 ± 7.4	104.1 ± 14.5	103.4 ± 20.1	.003
Postoperative platelets number (10^9/L)	105.7 ± 41.8	148.5 ± 62.8	118.2 ± 50.2	112.7 ± 35.2	.282
Postoperative prothrombin time (second)	12.3 ± 2.8	13.2 ± 2.5	13.5 ± 2.3	13.5 ± 1.9	.701
Postoperative APTT (second)	32.1 ± 5.2	33.5 ± 5.3	31.7 ± 4.5	32.3 ± 4.9	.892

### 3.5 The risk factors of massive blood transfusion

Logistic regression analysis suggests (Table 6) that the history of cesarean section (OR, 6.999, 95%CI: 2.441–20.070 and placenta previa (OR, 12.810, 95%CI: 3.036–54.045) were the main factors for red blood cell infusion >10 U.

**Table 6:** Multivariate logistic regression analysis for variables of red blood cell infusion >10 U.

							95% CI for EXP(B)	
	B	S.E.	Wald	df	*P*	Exp(B)	Lower	Upper
History of cesarean section	1.946	0.537	13.105	1	.000	6.999	2.441	20.070
Placenta previa	2.550	0.735	12.055	1	.001	12.810	3.036	54.045
Constant	−5.321	0.843	39.858	1	.000	0.005		

## 4 Discussion

The purpose of intraoperative blood transfusion is to correct blood loss, supply erythrocytes, improve blood oxygen-carrying capacity, improve microcirculation, supplement blood colloidal components and blood coagulation factors, and maintain normal coagulation functions. Common causes for infusion of blood products during cesarean section are prenatal coagulation deficiency of blood components and PPH. The main causes of prenatal deficiency include prenatal anemia, preeclampsia, AFLP, and prenatal blood loss (placental previa and placental abruption are common). PPH is a leading cause of maternal mortality, and is common in patients with postpartum uterine inertia and placental previa with or without placental implantation.^[13]^ In recent years, due to increasing use of second-line contraction drugs, uterine inertia induced PPH has been effectively controlled, while PPH caused by placenta previa and placental implantation has become more and more prominent.^[14]^

This study showed that total volume of blood transfusion and number of blood transfusion increased year by year from 2013 to 2016, especially in cases of major bleeding (≥1000 ml) requiring massive blood transfusion (≥10 U). The study of Jie in China also showed that total transfusion volume, consumed erythrocyte, and plasma volumes increased significantly in 2015 and 2016 with the launching of the two-child policy.^[15]^

While the trend of blood transfusion in the developed countries showed a decrease from 1996 to 2006.^[16]^ From our data analysis, causes of massive blood transfusions during cesarean section were 95.8% for placenta previa, and 93.1% for these who had history of cesarean section. Pernicious placenta previa refers to the occurrence of placenta previa with the placenta attached to the uterine scar. Its intraoperative bleeding is rapid and massive, and hemostasis is difficult, which usually requires massive blood transfusion, and even hysterectomy to control bleeding.^[11,12,17,18]^ Furthermore, PPH associated with pernicious placenta previa is extremely dangerous, which would lead to major bleeding, hemorrhagic shock, DIC, and severe complications of the surrounding bladder, ureters, or intestines.^[11,12]^ Cesarean section of pernicious placenta previa is a great challenge for obstetricians and anesthesiologist. Previous studies have shown that repeated cesarean section and placenta previa are independent risk factors for PPH, blood transfusion, and postpartum hysterectomy.^[19–22]^ Our findings also suggest that in the context of the two-child policy, cesarean section rate in primiparas needs to be controlled, for reducing the occurrence of scarred uterus and blood transfusion associated with repeated cesarean section as well.^[23]^

In the traditional concept of intraoperative blood transfusion, volume expansion with crystalloids should be performed firstly for patients with bleeding. When bleeding is greater than 20% of total blood volume of patients, erythrocyte infusion should be considered, and when bleeding is greater than 30%, the infusion of blood components such as erythrocytes, plasma, and cryoprecipitate should be considered.^[24]^ The traditional concept of blood transfusion is passive, conservative, and not timely, which is not suitable for patients with pernicious placenta previa who are expected to suffer from major bleeding.^[11,12]^ In recent years, studies in China and elsewhere have shown that blood infusion at an early stage for patients with acute major bleeding can greatly improve success of rescue and reduce mortality, and the concept of massive transfusion protocol (MTP) was proposed.^[25–27]^ It refers to the principle that blood transfusion is performed based on a pre-designed blood component protocol when major bleeding occurs, which requires active multidisciplinary collaboration of obstetrics, anesthesiology, and blood bank.

In this study, there was no significant difference in the ratio of erythrocyte infusion and plasma infusion for patients with massive blood transfusion during 2013 to 2016. The percentage of intraoperative platelet infusion increased year by year. Changes in coagulation functions should receive more attention in patients with massive blood transfusion in addition to infusion of erythrocytes and plasma in proportion. For massive blood transfusion, coagulation factors were supplemented by infusion of cryoprecipitate. Reasonable infusion of platelets and cryoprecipitate is especially important to maintain the coagulation functions of the patients. Nevertheless, because there are few sources of platelets, clinical application is delayed and conservative. After cesarean section, there were still patients with platelets <50 × 10^9^/L. Fortunately, there was no case of severe capillary hemorrhage and difficulty in hemostasis. The percentages of patients with postoperative coagulation abnormalities were 42.9%, 37.5%, 47.4%, and 40.0% in the year of 2013, 2014, 2015, and 2016, respectively. Although there were no serious complications such as DIC after cesarean section, the prolongation of PT and APTT, as well as the decrease of the fibrinogen were important issues that should be paid attention.

The importance of antifibrinolytic drugs is gradually recognized in improving coagulation functions in patients with major bleeding. In this study, there was only 1 case used tranexamic acid in 2014, while the tranexamic acid use rate in 2015 and 2016 increased significantly compared to the years of 2013 and 2014. Tranexamic acid is a synthetic lysine analog that binds to plasminogen in order to block its activation into plasmin. Tranexamic acid was developed in 1962 in the course of seeking medical treatment for PPH.^[28–30]^ An exploratory analysis of the CRASH-2 trial showed that the use of tranexamic acid at an early stage (<3 hours) can reduce the mortality due to bleeding in patients with trauma.^[31]^ Application of tranexamic acid in patients with PPH can also reduce the bleeding volume, potentially reducing blood transfusion and avoiding hysterectomy.^[32]^ On the other hand, the results of a World Maternal Antifibrinolytic trial showed that the use of tranexamic acid did not lead to an increase in thrombotic events,^[33]^ and the study recommended that it was reasonable to use tranexamic acid during severe PPH or when severe bleeding were expected to occur. At our center, tranexamic acid is generally administered intravenously at 1.0 g when bleeding ≥1000 ml based on the recommendations of previous references and guidelines,^[34–36]^ and there is risk of continuous bleeding, but no consensus in clinical practice for the exact use of tranexamic acid, which should be further investigated in clinical experiments. In this study, no patient suffered from thrombotic event after the use of tranexamic acid.

In this study, the difference in hemoglobin before and after cesarean section was statistically significant only in 2014. However, the average values of hemoglobin after the operation in 2015 and 2016 were slightly higher than those before the operation. Moreover, longitudinal comparison showed that hemoglobin in 2015 and 2016 was significantly higher compared to 2013. At first, blood transfusion was passive and the amount of blood transfusion was conservative to correct blood loss in patients with pernicious placenta previa due to limited awareness of the risk. After recognizing the risk of major bleeding to pernicious placenta previa, passive response was replaced by active response to major bleeding since the end of 2014. Therefore, postoperative hemoglobin can be maintained at a higher level.

The limitations of this study are that the patients were all from a single hospital, limiting the generalizability of the results. The 4 years is a relatively short period and the investigation time needs to be extended. Finally, this study is retrospective and therefore cannot analyze unrecorded factors in medical charts. In the future, we will focus on the cooperation of MDT to predict preoperative blood loss and strategies to reduce blood loss. The most important method is to reduce the rate of primary cesarean delivery to reduce the occurrence of morbidly adhesion of the placenta.

## 5 Conclusion

The two-child policy in China led to an increase popularity of repeat cesarean section. The frequency of placenta previa accompanied with previous cesarean section increased the need for massive blood transfusion. Our results indicate that the indications of cesarean section should be strictly limited to avoid unnecessary cesarean section for primipara. Repeat cesarean section is usually used for previous cesarean section. Therefore, it effectively reduces the dangerous placenta previa caused by uterine scars in limiting primary cesarean section, thereby reducing the rate of massive blood transfusion.

## Author contributions

**Conceptualization:** Changqing Zhou, Li Zhang, Yang Bao, Ling Li, Ting Zhang, Xiyan Zhang, Chunling Wang.

**Data curation:** Changqing Zhou, Li Zhang, Yang Bao, Ling Li, Ting Zhang, Xiyan Zhang, Chunling Wang.

**Formal analysis:** Changqing Zhou, Li Zhang, Yang Bao, Ling Li, Ting Zhang, Xiyan Zhang, Chunling Wang.

**Funding acquisition:** Changqing Zhou, Li Zhang, Yang Bao, Ling Li, Ting Zhang, Xiyan Zhang, Chunling Wang.

**Investigation:** Changqing Zhou, Li Zhang, Yang Bao, Ling Li, Ting Zhang, Xiyan Zhang, Chunling Wang.

**Methodology:** Changqing Zhou, Li Zhang, Yang Bao, Ling Li, Ting Zhang, Xiyan Zhang, Chunling Wang.

**Project administration:** Changqing Zhou, Li Zhang, Yang Bao, Ling Li, Ting Zhang, Xiyan Zhang, Chunling Wang.

**Resources:** Changqing Zhou, Li Zhang, Yang Bao, Ling Li, Ting Zhang, Xiyan Zhang, Chunling Wang.

**Software:** Changqing Zhou, Li Zhang, Yang Bao, Ling Li, Ting Zhang, Xiyan Zhang, Chunling Wang.

**Supervision:** Changqing Zhou, Li Zhang, Yang Bao, Ling Li, Ting Zhang, Xiyan Zhang, Chunling Wang.

**Validation:** Changqing Zhou, Li Zhang, Yang Bao, Ling Li, Ting Zhang, Xiyan Zhang, Chunling Wang.

**Visualization:** Changqing Zhou, Li Zhang, Yang Bao, Ling Li, Ting Zhang, Xiyan Zhang, Chunling Wang.

**Writing – original draft:** Changqing Zhou, Li Zhang, Yang Bao, Ling Li, Ting Zhang, Xiyan Zhang, Chunling Wang.

**Writing – review & editing:** Changqing Zhou, Li Zhang, Yang Bao, Ling Li, Ting Zhang, Xiyan Zhang, Chunling Wang.

## References

[1] Lee CR Lee JH Park KS Antimicrobial resistance of hypervirulent klebsiella pneumoniae: epidemiology, hypervirulence-associated determinants, and resistance mechanisms. Front Cell Infect Microbiol 2017;7:483. 29209595 10.3389/fcimb.2017.00483 PMC5702448

[2] Reducing the Global Burden: Postpartum Haemorrhage. Making Pregnancy Safer. Geneva: World Health Organization; 2007.

[3] Lutomski JE Byrne BM Devane D Increasing trends in atonic postpartum haemorrhage in Ireland: an 11-year population-based cohort study. BJOG 2012;119:306–14. 22168794 10.1111/j.1471-0528.2011.03198.x

[4] Jackson KW Jr Allbert JR Schemmer GK A randomized controlled trial comparing oxytocin administration before and after placental delivery in the prevention of postpartum hemorrhage. Am J Obstet Gynecol 2001;185:873–7. 11641669 10.1067/mob.2001.117363

[5] Sheiner E Sarid L Levy A Obstetric risk factors and outcome of pregnancies complicated with early postpartum hemorrhage: a population-based study. J Matern Fetal Neonatal Med 2005;18:149–54. 16272036 10.1080/14767050500170088

[6] Blomberg M Maternal obesity and risk of postpartum hemorrhage. Obstet Gynecol 2011;118:561–8. 21860284 10.1097/AOG.0b013e31822a6c59

[7] Hanley GE Smolina K Mintzes B Postpartum hemorrhage and use of serotonin reuptake inhibitor antidepressants in pregnancy. Obstet Gynecol 2016;127:553–61. 26855096 10.1097/AOG.0000000000001200

[8] Cheng PJ Duan T China's new two-child policy: maternity care in the new multiparous era. BJOG 2016;123: Suppl 3: 7–9. 27627588 10.1111/1471-0528.14290

[9] Weerasekera DS Placenta praevia and scarred uterus - an obstetrician's dilemma. J Obstet Gynaecol 2000;20:484–5. 15512632 10.1080/014436100434659

[10] 2016;Fan D Wu S Wang W Prevalence of placenta previa among deliveries in Mainland China: a PRISMA-compliant systematic review and meta-analysis Medicine (Baltimore). 95:e5107. 10.1097/MD.0000000000005107 PMC5059095 27749592

[11] Fan D Xia Q Liu L The incidence of postpartum hemorrhage in pregnant women with placenta previa: a systematic review and meta-analysis. PLoS One 2017;12:e0170194. 28107460 10.1371/journal.pone.0170194 PMC5249070

[12] Fan D Wu S Liu L Prevalence of antepartum hemorrhage in women with placenta previa: a systematic review and meta-analysis. Sci Rep 2017;7:40320. 28067303 10.1038/srep40320 PMC5220286

[13] Lozano R Naghavi M Foreman K Global and regional mortality from 235 causes of death for 20 age groups in 1990 and 2010: a systematic analysis for the Global Burden of Disease Study 2010. Lancet 2012;380:2095–128. 23245604 10.1016/S0140-6736(12)61728-0 PMC10790329

[14] Rani PR Begum J Recent advances in the management of major postpartum haemorrhage - a review. J Clin Diagn Res 2017;11:QE01–5. 10.7860/JCDR/2017/22659.9463 PMC5376787 28384942

[15] Jie Y Wei R Yu Y Data analysis of clinical transfusion in obstetrics. China Med Herald 2017;14:173–6.

[16] Pearson GA MacKenzie IZ Blood loss and blood transfusion at caesarean section: a prospective observational study covering 30 years. Eur J Obstet Gynecol Reprod Biol 2014;181:72–7. 25145760 10.1016/j.ejogrb.2014.06.025

[17] Chattopadhyay SK Kharif H Sherbeeni MM Placenta praevia and accreta after previous caesarean section. Eur J Obstet Gynecol Reprod Biol 1993;52:151–6. 8163028 10.1016/0028-2243(93)90064-j

[18] Xie X Gou W Obstetrics and Gynecology. Eighth Edition Beijing: People's Medical Publishing House; 2013.

[19] Ossola MW Somigliana E Mauro M Risk factors for emergency postpartum hysterectomy: the neglected role of previous surgically induced abortions. Acta Obstet Gynecol Scand 2011;90:1450–3. 21692756 10.1111/j.1600-0412.2011.01223.x

[20] Lu B Chen M Liu X Risk factors of peripartum hysterectomy in placenta previa: a retrospective study of 3840 cases. Chin J Obstet Gynecol 2016;51:498–502. 10.3760/cma.j.issn.0529-567X.2016.07.004 27465868

[21] Butwick AJ Ramachandran B Hegde P Risk factors for severe postpartum hemorrhage after cesarean delivery: case-control studies. Anesth Analg 2017;125:523–32. 28277324 10.1213/ANE.0000000000001962 PMC5522356

[22] Bao Y Xu C Qu X Risk factors for transfusion in cesarean section deliveries at a tertiary hospital. Transfusion 2016;56:2062–8. 27239976 10.1111/trf.13671

[23] Biler A Ekin A Ozcan A Is it safe to have multiple repeat cesarean sections? A high volume tertiary care center experience. Pak J Med Sci 2017;33:1074–9. 29142541 10.12669/pjms.335.12899 PMC5673710

[24] Liu J Zhao J Modern Anesthesiology. Second edition. Beijing: People's Medical Publishing House; 1999.

[25] Chambers LA Chow SJ Shaffer LE Frequency and characteristics of coagulopathy in trauma patients treated with a low- or high-plasma-content massive transfusion protocol. Am J Clin Pathol 2011;136:364–70. 21846911 10.1309/AJCPH16YXJEFSHEO

[26] Tan JN Burke PA Agarwal SK A massive transfusion protocol incorporating a higher FFP/RBC ratio is associated with decreased use of recombinant activated factor VII in trauma patients. Am J Clin Pathol 2012;137:566–71. 22431532 10.1309/AJCPQZNCHM5PIK8O

[27] Sinha R Roxby D Bersten A Experience with a massive transfusion protocol in the management of massive haemorrhage. Transfus Med 2013;23:108–13. 23521624 10.1111/tme.12022

[28] Butwick AJ Deneux-Tharaux C Sentilhes L Tranexamic acid for the management of obstetric hemorrhage. Obstet Gynecol 2017;130:1386. 10.1097/AOG.0000000000002384 29189683

[29] Sullivan JT The expanding role of tranexamic acid in the management of obstetric hemorrhage. J Thorac Dis 2017;9:2251–4. 28932517 10.21037/jtd.2017.07.19 PMC5594136

[30] Pacheco LD Hankins GDV Saad AF Tranexamic acid for the management of obstetric hemorrhage. Obstet Gynecol 2017;130:765–9. 28885402 10.1097/AOG.0000000000002253

[31] CRASH-2 collaborators Roberts I Shakur H The importance of early treatment with tranexamic acid in bleeding trauma patients: an exploratory analysis of the CRASH-2 randomised controlled trial. Lancet 377:1096–101. 1101.e1-2. 10.1016/S0140-6736(11)60278-X 21439633

[32] Heesen M Bohmer J Klohr S Prophylactic tranexamic acid in parturients at low risk for post-partum haemorrhage: systematic review and meta-analysis. Acta Anaesthesiol Scand 2014;58:1075–85. 25069636 10.1111/aas.12341

[33] Collaborators WT Effect of early tranexamic acid administration on mortality, hysterectomy, and other morbidities in women with post-partum haemorrhage (WOMAN): an international, randomised, double-blind, placebo-controlled trial. Lancet 2017;389:2105–16. 28456509 10.1016/S0140-6736(17)30638-4 PMC5446563

[34] Ducloy-Bouthors AS Jude B Duhamel A High-dose tranexamic acid reduces blood loss in postpartum haemorrhage. Crit Care 2011;15:R117. 21496253 10.1186/cc10143 PMC3219400

[35] Novikova N Hofmeyr GJ Tranexamic acid for preventing postpartum haemorrhage. Cochrane Database Syst Rev 2010 CD007872. 20614466 10.1002/14651858.CD007872.pub2

[36] Peitsidis P Kadir RA Antifibrinolytic therapy with tranexamic acid in pregnancy and postpartum. Expert Opin Pharmacother 2011;12:503–16. 21294602 10.1517/14656566.2011.545818

